# Therapeutic Potential of Extracellular Vesicles in Aging and Age-Related Diseases

**DOI:** 10.3390/ijms232314632

**Published:** 2022-11-23

**Authors:** Jorge Sanz-Ros, Cristina Mas-Bargues, Nekane Romero-García, Javier Huete-Acevedo, Mar Dromant, Consuelo Borrás

**Affiliations:** 1 Freshage Research Group, Department of Physiology, Faculty of Medicine, University of Valencia, Centro de Investigación Biomédica en Red Fragilidad y Envejecimiento Saludable-Instituto de Salud Carlos III (CIBERFES-ISCIII), INCLIVA, 46010 Valencia, Spain; 2 Cardiology Department, Hospital Universitari i Politècnic La Fe, 46026 Valencia, Spain

**Keywords:** intercellular communication, extracellular vesicles, stem cells, age-related diseases

## Abstract

Aging is associated with an alteration of intercellular communication. These changes in the extracellular environment contribute to the aging phenotype and have been linked to different aging-related diseases. Extracellular vesicles (EVs) are factors that mediate the transmission of signaling molecules between cells. In the aging field, these EVs have been shown to regulate important aging processes, such as oxidative stress or senescence, both in vivo and in vitro. EVs from healthy cells, particularly those coming from stem cells (SCs), have been described as potential effectors of the regenerative potential of SCs. Many studies with different animal models have shown promising results in the field of regenerative medicine. EVs are now viewed as a potential cell-free therapy for tissue damage and several diseases. Here we propose EVs as regulators of the aging process, with an important role in tissue regeneration and a raising therapy for age-related diseases.

## 1. Introduction

Stem cell therapy has been the leading framework in regenerative medicine. Owing to their self-renewal capacity, exogenous stem cells should be able to regenerate different tissues after damage. Mesenchymal stem cells (MSCs), due to their easy isolation, culture, and low immunogenicity, have been the most studied type of cells for their application in a clinical setting [1]. Historically, the main hypothesis was that stem cells should have the ability to integrate into damaged tissue and repopulate it through asymmetric division. However, more recent studies have shown that allogeneic MSCs have a limited potential for integration into a tissue, as most commonly, less than 1% of the transferred cells survive more than a week in the host organism [2,3,4,5]. Despite these facts, MSCs therapies have been demonstrated to be beneficial for many conditions and improve the regeneration of several tissues, suggesting that the most significant effects of these cells are mediated by paracrine mechanisms [6,7]. Thus, the secretome of MSCs has been positioned as a potential therapy for tissue repair and regeneration.

Among all the factors that stem cells release to the extracellular environment, both in vivo and in vitro, extracellular vesicles (EVs) have emerged as a very interesting choice. These lipid bilayer vesicles are released by virtually every cell type and contain a myriad of molecules that are thought to be responsible for their role in the communication between cells, through the exchange of proteins, nucleic acids, or lipids [8,9]. There are many types of EVs, such as exosomes, microvesicles, ectosomes, oncosomes, or apoptotic bodies, these are classified attending to their biological origin and role [8]. However, this classification is not the most convenient due to the lack of methods that can separate specifically the different subtypes of EVs. The most used methods for isolating and separating EVs rely on their size (i.e., differential ultracentrifugation, filtration, size exclusion chromatography, or high molecular weight filters). Other methods are based on the differences in EVs density (density gradient) or specific surface molecules (immunoaffinity isolation techniques). The International Society for Extracellular Vesicles proposes a classification based on the size of the vesicles, distinguishing small extracellular vesicles (<200 nm in diameter) from other medium and larger vesicles [9]. Although small extracellular vesicles are probably the most studied, here we will describe findings related to all types of EVs, regardless of the nomenclature used by the authors of each study.

The use of EVs adds several advantages over the use of stem cells to improve tissue regeneration. EVs are more stable, their dosage is easier, they do not have a risk of aneuploidy, and they have a lower incidence of immune rejection [7]. EVs from several types of stem cells have demonstrated their potential to improve regeneration in many different tissues after induced damage, such as the kidney, liver, heart, or brain [10].

Aging is characterized by a loss of tissue regenerative capacity. All the stem cells’ niches that are present in an adult organism lose the ability to regenerate properly, both in basal conditions and after damage, with a lower number of potential divisions over a longer period [11]. As aging progresses, progenitor cells responsible for damaged tissue regeneration suffer molecular traits that affect their ability to replicate, such as DNA damage accumulation, telomere attrition, or senescence [12]. EVs from stem cells and other cell types are now being studied as potential regulators of many processes associated with aging, such as cellular senescence, oxidative stress, telomere dysfunction, autophagy inefficiency, inflammation, and metabolic dysregulation.

In this review, we aim to point out the idea that EVs from different sources such as stem cells could be beneficial for the aging-associated decline in tissue function and age-related diseases, aiding tissue regeneration through different pathways.

## 2. Role of EVs in the Aging Process

**Figure 1:**
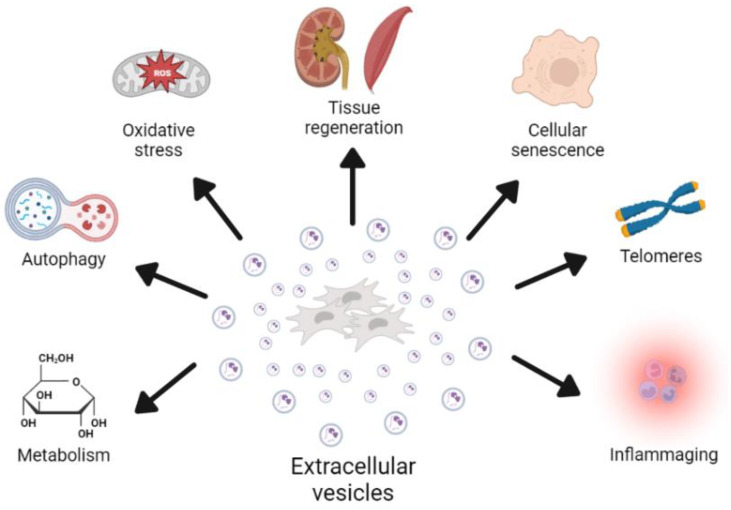
Graphical explanation of the role of EVs in the modulation of multiple processes associated with aging. The concrete effects of these processes are described in the manuscript, and EVs have been shown to participate in both directions. Depending on cell source and condition, EVs have beneficial or deleterious effects on age-related processes.

Aging is characterized by molecular and cellular traits that are thought to be the drivers of this process, which ultimately lead to tissue dysfunction and an increased risk of death [12,13]. EVs from different cells have been described as factors that can modify these aging-associated processes (Figure 1). Here, we will describe some of the last findings regarding the role of EVs in aging.

### 2.1. Cellular Senescence

Cellular senescence is a physiological process that some cells suffer when exposed to different types of damage, such as DNA damage, oncogene activation, telomere attrition, or radiation [14]. These cells enter a cell cycle arrest that prevents their proliferation as a tumor suppressor mechanism. However, the accumulation of these cells during aging, due to increased production and a decreased clearance by the immune system, has been linked to several aging processes and pathologies [15]. An important contributor to the deleterious effect of senescent cells is the accumulation of different factors in the extracellular milieu, the senescence-associated secretory phenotype (SASP) [16]. These factors have been described as drivers of paracrine senescence, cancer growth, and inflammation, among others [17]. As players in intercellular communication, EVs are now regarded as important factors of the SASP [18], with an important role in the deleterious effects of senescence, referred to as evSASP [19,20]. The release of EVs is typically increased in senescent cells, and its content changes when cells become senescent, suggesting an important mechanism to target in cellular senescence [20,21].

Some studies have shown a promising therapeutic effect of EVs from healthy, non-senescent cells, such as stem cells, in the modulation of senescence. EVs from healthy cells can decrease senescence both in vivo and in vitro [22,23,24] through several proposed mechanisms, such as the transmission of miRNAs [22,24], antioxidant enzymes [23], or metabolic changes [25]. Due to their immunomodulatory roles, EVs can act through the modulation of the SASP, decreasing the pro-inflammatory changes in the microenvironment induced by the accumulation of senescent cells. One of the proposed mechanisms that might explain the effects of EVs on senescence is viewing EVs from healthy cells as senomorphics, factors that can decrease the burden of senescent cells, not by directly inducing their selective apoptosis as senolytics but by modifying the extracellular environment and inhibiting the SASP [24,26].

### 2.2. Oxidative Stress

Oxidative stress, and more concretely, the fine control of the redox status of a cell or a tissue, is thought to contribute to the aging process and age-related morbidities [27,28]. The balance between oxidative and reductive molecules is crucial to the proper functionality of the cells. During aging, dysregulation of these control mechanisms occurs, with some tissues tending to accumulate oxidative damage in several macromolecules, mainly lipids, proteins, or DNA. This damage contributes to the development of age-related pathologies, such as Alzheimer’s [29] or frailty [30,31]. EVs are now viewed as important factors that regulate the redox status through the transfer of molecules with antioxidant properties between cells, protecting against oxidative damage in several models of acute damage and during aging [23,24,32]. The different conditions associated with the culture of cells can modify EVs release, and the content of the oxygen tension in the culture is of the utmost importance [33,34]. EVs from cells cultured under a physiological oxygen tension show increased therapeutic effects in some models of tissue damage, with changes in their content and release, suggesting that oxygen availability is an important mechanism that modulates EV biogenesis and content [35,36].

### 2.3. Telomere Dysfunction

Telomere shortening and damage, as well as dysregulation of the machinery that controls the structure of telomeres, are important players in the aging process and are tightly related to other processes of aging, such as senescence or genomic instability [37,38]. In these terms, EVs have been shown to contain short non-coding telomere RNA transcripts that can induce the release of inflammatory cytokines in recipient cells, linking telomere dysfunction and inflammation through EV release [39]. EVs from stem cells have been shown to increase telomerase activity in a model of osteoporosis in mice. Moreover, EVs have been described as factors that regulate telomere-related bystander effects in irradiated breast cancer cells. EVs from irradiated cells decreased telomerase activity in recipient cells, with shorter telomeres over time in these cells [40]. RNAse treatment suppressed these effects, suggesting the role of RNAs contained in EVs in the regulation of telomere function. In a more recent study, telomere vesicle transfer from antigen-presenting cells elongated telomeres in recipient T cells independently of telomerase activity [41].

### 2.4. Autophagy

The correct regulation of autophagy is key to maintaining proteostasis during aging, and a loss of this regulatory mechanism has been linked to aging and age-related diseases [42,43]. During aging, there is an accumulation of damaged components of the proteome, both intracellular and extracellular, such as misfolded or aggregated proteins [12]. Trying to improve autophagy signaling is an interesting approach to treating age-related diseases, such as Parkinson’s or Alzheimer’s [44]. Autophagy is closely related to the biogenesis of extracellular vesicles, as exosomes are included in multivesicular bodies before their release [8], and some autophagy-related proteins, such as ATG5, have been shown to regulate exosome biogenesis [45]. EVs from different types of MSCs have been demonstrated to promote the autophagic flux in several disorders associated with aging. In a model of diabetic nephropathy, EVs from ADSCs inhibited excessive mTOR activation, leading to increased autophagy [46,47]. A similar effect of improved outcomes due to autophagy activation has been observed in liver fibrosis [48] and spinal cord injury [49]. Therefore, the modulation of autophagy by EVs, and vice versa, may serve as a therapeutic tool in age-related conditions and the maintenance of proteostasis.

### 2.5. Inflammation

One of the most studied effects of EVs is their potential role in regulating the immune system, as EVs from damaged cells usually induce an activation of the immune system, leading to the release of pro-inflammatory factors [50]. On the other hand, EVs from MSCs are now viewed as significant anti-inflammatory and immunomodulatory factors [51,52]. MSCs themselves have been studied for a long time as promising therapeutics for inflammatory diseases, as they have been shown to decrease the pro-inflammatory activity of several cell types, such as neural, vascular, or osteochondral [53]. During aging, an imbalance between pro and anti-inflammatory factors occurs, leading to an accumulation of pro-inflammatory factors in the extracellular environment that accompanies normal aging and that has been linked to several age-related diseases [54]. This chronic inflammation has been termed “inflammaging”, and the interventions targeting this process have beneficial effects for age-related conditions [55,56]. As stated before, the beneficial effect of EVs observed in many models of disease, such as myocardial infarction, stroke, chronic kidney disease (CKD), or liver injury, is typically accompanied by a suppression of pro-inflammatory cytokines, with an increase in anti-inflammatory molecules. In physiological aging, the treatment with EVs has been shown to decrease inflammatory cytokines that are associated with aging, such as IL-6, IL-1β, or TNF-α [24,26,57,58]. As we have described, EVs are important factors of the SASP that lead to paracrine senescence and contribute to the chronic inflammation observed during aging [20]. One of the proposed mechanisms through which EVs from healthy cells could decrease inflammation and senescence is the inhibition of the SASP, acting as “senomorphics”, linking their effect both in cellular senescence and inflammation [24,26].

### 2.6. Metabolism

One of the most conserved mechanisms in evolution that controls aging and longevity is metabolism, mainly through several pathways related to nutrient sensing and growth, such as insulin/IGF-1, mTOR, AMPK, or sirtuins [59,60,61,62]. In this sense, manipulations that target these pathways have been demonstrated to have a wide effect on longevity and health span (i.e., caloric restriction or mTOR inhibitors) [63,64,65]. EVs have been shown to induce profound metabolic changes in recipient cells; for example, EVs from obese mice can increase insulin resistance and dyslipidemia in healthy mice through the transfer of miRNAs [66,67]. Moreover, miRNAs that suffer age-associated changes in plasmatic EVs are important regulators of these metabolic pathways [68]. As aging progresses, the content of nicotinamide phosphoribosyltransferase (NAMPT) in circulating EVs decay, and the restoration of circulating levels of NAMPT have shown to improve health span and extend lifespan in mice, mainly through the increase in NAD+ biosynthesis in old tissues [25]. In another study, authors observed that plasmatic EVs from young mice induce a decrease in mTOR and IGF1R in the liver and lungs of old mice [58]. All these data suggest that EVs play a role in metabolic regulation, with important effects on age-related pathways.

## 3. Pro-Regenerative Effects of EVs

Aging is characterized by decreased tissue repair and regeneration; thus, we propose that EVs may be useful for treating diseases associated with anomalous repair after damage. EVs from different sources have been applied in preclinical models of tissue damage. The most used source in these studies is MSCs, probably due to their simpler isolation and culture techniques. The site of EV injection varies between studies, with the majority injecting them intravenously or at the site of the damage whenever possible. Here we will briefly describe the effects on several damaged tissue models of various types of EVs. A summary of the pathways involved in the beneficial effects of EVs in tissue regeneration is shown in Table 1.

### 3.1. Effects of EVs on the Nervous Tissue

Nervous tissue has a limited regenerative capacity upon several types of damage, both in peripheral nervous tissue and in the central nervous system [82]. Stem cell-derived EVs have been shown to improve regeneration of the nervous tissue after some types of damage in several animal models, such as peripheral nerve denervation [83,84], trauma [85], and stroke [86,87], with a neuroprotective effect on models of hypoxic-ischemic encephalopathy [69,88]. These EVs seem to exert this role through different processes and pathways associated with nerve regeneration. For example, in a recent study, Turovsky et al. demonstrated that EVs from MSCs exert a neuroprotective effect in a mouse model of ischemia through the modulation of phosphatidylinositol 3-Kinase/AKT signaling and calcium oscillations [69]. Moreover, EVs from different sources can induce changes in the glial cells, increasing the percentage of proliferating Schwann cells by 20% and increasing the myelinating potential of oligodendrocytes [89]. EVs also induce protective effects on the neurons themselves, improving neurogenesis and angiogenesis [87], doubling neurite length and increasing branch number by 75% [86], and reducing neuron apoptosis and neuroinflammation [70].

### 3.2. Effects of EVs on the Cardiovascular System

The models used to test the regenerative effects of EVs from different types of stem cells have been mainly models of myocardial infarction and ischemia-reperfusion injury. In these studies, EVs have been demonstrated to improve cardiac tissue repair, inhibiting the adverse remodeling that typically occurs after myocardial ischemia [71,90]. Cardiomyocytes are post-mitotic cells that do not regenerate after damage; the presence of resident cardiac stem cells in heart tissue is still debated [91]. EVs promote the regeneration of tissue through different pathways. It has been described that these EVs improve cardiomyocyte viability, reduce infarct size by 50% [71], decrease the apoptosis of these cells [92], and increase the capacity to revascularize the damaged tissue through improved angiogenesis [72]. EVs encapsulated in hydrogels have been demonstrated to decrease the excessive fibrosis and upregulation of pro-inflammatory factors, which are linked to a defective repair of cardiac tissue [93].

Endothelial cells are involved in the vascularization of every organ, and adequate angiogenesis is crucial to the regenerative process [94]. In this regard, EVs from stem cells and other cell types have been linked to improved angiogenesis in many tissue damage models, thus increasing the tissue regeneration potential of the tissue [73].

### 3.3. Effects of EVs on the Musculoskeletal System

EVs from several types of stem cells have been demonstrated to improve the regeneration of different tissues of the musculoskeletal system. EVs from muscle progenitor cells improve myogenesis both in vitro and in vivo [95]. In muscle damage models, such as ischemia [96], torn rotator cuffs [97], or muscle laceration [74], EVs can improve regeneration through increased angiogenesis, satellite cell activation [74], and decreased inflammation and fibrosis [95]. In the field of aging, EVs from the serum of young mice have shown an effect on improving muscle regenerative capacity in a model of cardiotoxin damage in old mice [75]. EVs from MSCs can improve and accelerate bone healing in models of fracture [98] or radiation-induced bone damage [76], inducing osteogenic cell proliferation and bone angiogenesis, leading to an increase in new bone area formation of 40% [77,99]. Similar effects have been observed in models of cartilage damage, where EVs from MSCs improve cartilage regeneration when injected intraarticularly [100].

### 3.4. Effects of EVs on Damaged Lungs

Some models of acute lung injury, such as hyperoxia [101], severe pneumonia [102], or endotoxin [103], have been developed to test the effect that EVs from stem cells have on lung tissue regeneration and repair. In these studies, EVs from different sources have been demonstrated to improve tissue function, reducing inflammation and edema [104], oxidative stress, senescence, and fibrosis [78,79]. More specifically, EVs attenuated pulmonary fibrosis by 33% in a model of bleomycin-induced fibrosis, ameliorating myofibroblast division and collagen accumulation and inducing structural changes in the alveolar structure [79].

### 3.5. Effects of EVs on Damaged Kidney

The functional unit of kidneys, the nephron, is not able to fully regenerate after damage in adult kidneys, as they cannot form new glomeruli [105]. However, tubular epithelial cells can repopulate the damaged tissue after damage [106]. EVs from stem cells have been shown to improve kidney regeneration and function in some models of acute kidney injury (AKI), such as ischemia-reperfusion injury [107,108] or nephrectomy [109]. An increase in epithelial tubular cell proliferation and viability has been described [80,110], as well as a decrease in apoptosis, oxidative stress, and inflammation [80]. All these factors improved the recovery of renal function in these models. Other groups have studied the effect not only in AKI but also in chronic kidney damage. Stem cell-derived EVs have been shown to preserve renal functioning, improving cell survival through inhibition of profibrotic genes and apoptosis, showing a 30% decrease in BUN (blood urea nitrogen) in a mouse model of cyclosporin damage [111].

### 3.6. Effects of EVs on Damaged Liver

The adult liver is one of the tissues that are typically able to restore organ function after damage. Hepatocytes are active proliferating cells that can repopulate the tissue completely. However, hepatic failure can occur if the regenerative process is not adequate. EVs from different cells have been shown to have protective effects on liver damage, showing a decrease of around 40% in serum transaminases levels. EVs promote hepatocyte proliferation in drug-induced damage [112] and ischemia-reperfusion [81,113], decreasing apoptosis, oxidative stress, and excessive inflammation [114]. Similarly to other tissues, EVs decrease fibrosis progression in models of hepatic fibrosis [115].

## 4. Therapeutic Potential of EVs in Age-Related Diseases

**Figure 2:**
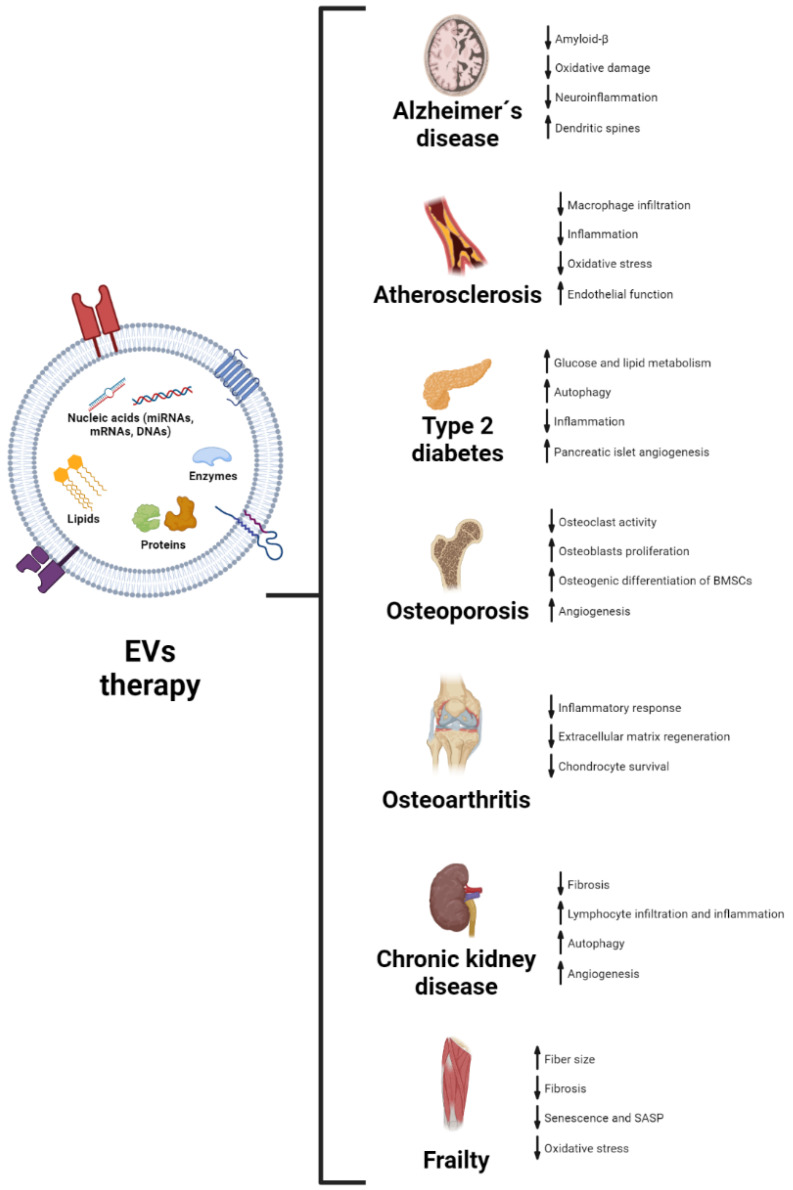
Recapitulation of the effects of treatment with EVs in preclinical models of age-associated diseases. For each condition, observed effects of EVs on different biological processes are shown, with an arrow indicating the increase or decrease in a concrete process. BMSCs: bone marrow-derived stem cells; SASP: senescence-associated secretory phenotype.

As we have explained, EVs from different sources have an important role in several cellular and molecular processes associated with aging, even reversing some of these markers both in vivo and in vitro. Hence, it is fair to propose that EVs may have a therapeutic role in age-related diseases. The role of EVs in the pathophysiology of these diseases, along with their potential use as biomarkers for these, has been reviewed before [116,117,118]. Here, we will focus on some of the most relevant studies using EVs as a therapeutic tool in age-related conditions. A summary of the results described is shown in Figure 2.

### 4.1. Alzheimer’s Disease

EVs from healthy cells, mainly stem cells, are now being studied as potential therapeutics in AD. Using transgenic mice that develop Alzheimer’s, multiple studies have shown that EVs from neural and mesenchymal stem cells can rescue cognitive decline associated with AD [119,120]. EVs reduce several markers of the disease, such as amyloid-β accumulation [120], oxidative damage [121], neuroinflammation, and microglia activation [122], along with an increase in dendritic spines. These studies have offered some candidate factors included in EVs that could be responsible for the effect, mainly miRNAs that are upregulated in EVs from stem cells [123].

### 4.2. Atherosclerosis

Atherosclerosis development in different parts of the vasculature is a common feature of aging, and it has a major effect on the decline of tissue function associated with aging, being one of the most important contributors to cardiovascular disease [124]. Many drugs target the accumulation of lipids and slow the progression of the disease; however, we lack a disease-modifying treatment for atherosclerosis [125]. EVs from different cell types, such as MSCs [126] or platelets [127], have shown promising effects on the treatment of atherosclerosis and plaque development. These EVs have been shown to influence the behavior of cells associated with atherosclerosis; for example, MSCs-derived EVs can reduce macrophage infiltration through miRNA delivery [128], with anti-inflammatory effects on eosinophils and endothelial cells (ECs) [129,130]. In another study, the authors demonstrated that EVs and associated miRNAs from endothelial progenitor cells can target ECs in atherosclerotic plaques, reducing oxidative stress, inflammation, and endothelial contractile dysfunction [131].

### 4.3. Type 2 Diabetes

Another relevant disease associated with aging and a contributor to the development of many age-related conditions is type 2 diabetes (T2DM) [132]. Altered glucose and other macromolecule metabolism is key in the pathogenesis of diabetes, something that is also altered with aging [62]. Researchers have studied the effect of EV treatment on some preclinical models of the disease. EVs from different sources, mainly MSCs, have shown therapeutic potential in T2DM [133]. The main effects are improved glucose and lipid metabolism, induction of autophagy [134], and inhibition of inflammatory response [135], all leading to improved insulin sensitivity. EVs from other sources, such as endothelial progenitor cells [136] or pancreatic β cells [137], have been shown to induce angiogenesis in pancreatic islets, promoting the survival of β cells and their function.

### 4.4. Osteoporosis

Osteoporosis incidence rises as we age and is particularly abundant in the post-menopausal female population [138]. The main model used in mice to study osteoporosis is the induction of post-menopausal osteoporosis through ovariectomy in female mice. This model has been used to test the effect of EVs from different sources in the development of osteoporosis. Due to its close association with the bone, bone marrow-derived stem cells (BMSCs) are the most studied source of EVs for osteoporosis treatment; other interesting sources are serum and milk. EVs from these sources have shown a beneficial effect on the progression of osteoporosis [139,140]. The proposed mechanisms are the inhibition of osteoclasts resorption activity [141], an induction of osteogenesis through increased osteoblasts proliferation and activity [139,142,143], promotion of the osteogenic differentiation of BMSCs [143], and an increase in vascularization via angiogenesis [144]. Some proteins that regulate osteogenesis, such as CLEC11A or MALAT1, are present in EVs and have been proposed as potential factors implicated in the beneficial effect [140,145]. Other proposed factors are miRNAs included in EVs that can modulate different pathways associated with osteoporosis development [142,144].

### 4.5. Osteoarthritis

One of the most common causes of disability in developed countries is osteoarthritis (OA). Nearly every person develops some degree of OA throughout their lives, with few therapeutic options apart from orthopedic treatment and pain management [146,147]. The main risk factor for OA is aging, so targeting this process is now an approach to modifying the natural history of OA [146]. SCs are the main source of EVs studied for the treatment of OA, as intra-articular and systemic injections of different SCs populations have been an attractive field in regenerative medicine due to their immunomodulatory properties that reduce excessive inflammation in OA [148]. EVs from MSCs have demonstrated their potential as therapeutics in preclinical models of the disease, mainly surgical models and intra-articular injection of different toxins [149]. They have shown beneficial effects on extracellular matrix regeneration [150], promotion of chondrocyte survival [149], as well as immunomodulatory effects [151]. Some proteins [152], miRNAs [153,154], and even glycans [155] that are present in MSCs-derived EVs are proposed molecules with therapeutic effects by themselves and are now being studied as therapeutics for OA.

### 4.6. Chronic Kidney Disease

Aging is usually accompanied by a progressive decline in renal function, and the incidence of CKD and renal failure rises as people age [156]. As we have stated before, EVs have been widely studied in kidney regeneration, especially those coming from MSCs. There are preclinical models of CKD in which EVs have been studied, mainly toxic induction of CKD [157], remnant kidney model [109], unilateral ureter obstruction [158,159], and diabetes [160]. EVs from MSCs have exhibited effects on a large number of processes associated with the development of CKD, with an effect not only on these processes but in renal function determined by serum urea and creatinine. These effects are inhibition of fibrosis [157], decrease in lymphocyte infiltration and inflammatory markers [109], induction of autophagy [47], and angiogenesis [161].

### 4.7. Frailty

Per definition, frailty is the physiological state characterized by an increase in the vulnerability to external aggressions because of a decrease in the physiological reserves of several systems [162]. Frailty prevalence increases dramatically with age and is one of the most accurate predictors of dependence and mortality in old people [163]. The search for therapies to improve the decline in physical performance and to prevent sarcopenia is particularly attractive in the aging field. In the last year, we and others have demonstrated an effect of EVs from different sources in the muscle of old mice. Sahu et al. showed that circulating EVs in the plasma of young mice were able to rejuvenate old muscle cells and improve the muscle regenerative capacity of old mice [75], leading to an increase in fiber size, muscle force, and mitochondriogenesis, with a decrease in fibrosis. They proposed that Klotho transcripts (which decline with age) present in young EVs were the main factors responsible for the effects observed. More recently, we have evidenced a beneficial effect of EVs from young adipose-derived MSCs in the physical performance of old mice, as well as a decrease in frailty, these effects were accompanied by an increase in fiber size and muscle protein content, with a decrease in muscle senescence and SASP factors, oxidative stress and lipid deposition [24].

## 5. Overview

Aging is the most important factor for the development of many diseases that affect us, which are the main cause of disability and mortality in almost every country [164]. The use of therapeutics that target the causes of aging at a cellular and molecular level is one of the most promising fields of study in medicine [13,165]. We will probably need to target several of these processes to treat age-related diseases. In this sense, EVs have been demonstrated to regulate a wide range of cellular processes due to their capacity to mediate intercellular communication [8,166].

As we have exposed in this work, EVs from different sources, particularly those released by stem cells, due to their pro-regenerative and immunomodulatory properties, are presented as a cell-free therapy that has demonstrated substantial effects in preclinical models of several age-related diseases and tissue regeneration. EVs not only have beneficial effects on diseases, but they have also been shown to regulate many processes associated with aging, with remarkable effects on the regenerative capacity of the tissues. In fact, in the last years, EVs from young mice and healthy cells have proven to recapitulate some of the effects of already proven interventions in aging, such as parabiosis, dietary restriction, mTOR inhibition, or senolytics [23,24,25]. Although these processes seem to be partly regulated by EVs, the different sources of EVs are also subjected to the aging process. As we have shown, the age of the donor is key to the beneficial effect of EVs [24]. The content in EVs differs between young and old cells. As cells become old, they start to release factors to the extracellular environment that result in deleterious effects such as paracrine senescence [19,20]. Thus, when developing therapeutics for age-related conditions, the use of young cells or plasma would probably bring better results. Regarding this, we still do not know the optimal source of EVs. Adult stem cells (mainly MSCs) seem to be a reasonable source for all the aforementioned properties. However, future studies should elucidate if other types of SCs, such as embryonic SCs or induced pluripotent SCs, have a greater potential in targeting the aging process.

The use of EVs as therapy by themselves is now being studied in clinics, with some clinical trials ongoing [167]. However, we still need to keep studying the basic biology of EVs and improving isolation and manufacturing methods to arrive at real patients. It is clear that we still need standardization protocols for the production, isolation, and manufacturing of EVs, as well as a need to understand the mechanisms of action before we can arrive at a feasible use of EVs in clinics [9,168]. There are two more alternatives to the use of whole EVs. The first is to use EVs as drug delivery vehicles, adding an interesting feature to the beneficial effect of EVs, with some studies showing the superiority of EVs loaded with different drugs or factors compared with EVs alone [169].

The second avenue of research is to explore EVs content and find different molecules or combinations of molecules that can recapitulate the beneficial effects observed with EVs treatments. This approach would probably result in a faster and simpler way for the clinics. In this regard, a great number of proteins, miRNAs, and lipids have been proposed in different studies. In preclinical studies, miRNAs are probably the most studied factors, as they can regulate many different pathways; however, some studies suggest that miRNAs are minor constituents of EVs with very low capacity to enter target cells [170,171].

Therefore, there is still a need for studies on the basic biology of EVs and the mechanisms that mediate both the release from donor cells and the effects on recipient cells. Nevertheless, we believe that both EVs and their content are interesting therapeutic tools in aging and age-related diseases, thanks to their ability to regulate many features associated with the aging process.

## Figures and Tables

**Table 1:** Outline of the pathways involved in the pro-regenerative effects of EVs in different tissues. PI3K: phosphatidylinositol-3-kinase; AKT: protein kinase B; WNT: wingless-related integration site; JNK: c-Jun N-terminal kinase; ATM: ATM serine/threonine kinase; IGF: insulin-related growth factor; HGF: hepatic growth factor; FGF: fibroblast growth factor; mTOR: mammalian target of rapamycin; RPTOR: regulatory-associated protein of mTOR; TGF-β: transforming growth factor β; CCL2: chemokine (C-C motif) ligand 2.

References	Tissue	Effect	Pathway Involved
[69]	Brain	Neuroprotective effect in ischemia and traumatic brain injury	Activation of PI3K/AKT pathway and calcium oscillations
[70]	Brain	Decrease in neuronal apoptosis and motor recovery	Activation of Wnt/β-catenin signaling
[71]	Heart	Enhanced myocardial viability in ischemia/reperfusion	Activation of PI3K/AKT pathway and reduced phosphorylated-c-JNK
[10,72,73]	Endothelial cells	Improved angiogenesis	Activation of VEGF signaling and PI3K/AKT, repression of ATM
[74]	Muscle	Prevention of muscle damage in hind limb ischemia	Activation of Neuregulin-1
[74]	Muscle	Improved myogenesis	Increased IGFs, HGFs and FGFs
[75]	Muscle	Improved muscle regeneration in aging	Activation of Klotho pathway
[76]	Bone	Decrease in radiation-induced bone loss	Activation of Wnt/β-catenin signaling
[77]	Bone	Improved bone regeneration	Activation of AKT/mTOR pathway
[78]	Lung	Amelioration of acute lung injury	Induction of RPTOR/mTOR pathway
[79]	Lung	Antifibrotic effect in a model of pulmonary fibrosis	Inhibition of TGF-β-Wnt crosstalk
[80]	Kidney	Decreased kidney injury in ischemia/reperfusion	Inhibition of CCL2 pathway
[81]	Liver	Amelioration of hepatic ischemia/reperfusion injury	Inhibition of MEK/ERK pathway

## References

[1] Han Y., Li X., Zhang Y., Chang F., Ding J. (2019). Mesenchymal Stem Cells for Regenerative Medicine. Cells.

[2] Müller-Ehmsen J., Whittaker P., Kloner R.A., Dow J.S., Sakoda T., Long T.I., Laird P.W., Kedes L. (2002). Survival and development of neonatal rat cardiomyocytes transplanted into adult myocardium. J. Mol. Cell. Cardiol..

[3] Toma C., Wagner W.R., Bowry S., Schwartz A., Villanueva F. (2009). Fate of culture-expanded mesenchymal stem cells in the microvasculature: In vivo observations of cell kinetics. Circ. Res..

[4] Eggenhofer E., Benseler V., Kroemer A., Popp F.C., Geissler E.K., Schlitt H.J., Baan C.C., Dahlke M.H., Hoogduijn M.J. (2012). Mesenchymal stem cells are short-lived and do not migrate beyond the lungs after intravenous infusion. Front. Immunol..

[5] Lee R.H., Pulin A.A., Seo M.J., Kota D.J., Ylostalo J., Larson B.L., Semprun-Prieto L., Delafontaine P., Prockop D.J. (2009). Intravenous hMSCs improve myocardial infarction in mice because cells embolized in lung are activated to secrete the anti-inflammatory protein TSG-6. Cell Stem Cell.

[6] Chen L., Tredget E.E., Wu P.Y., Wu Y. (2008). Paracrine factors of mesenchymal stem cells recruit macrophages and endothelial lineage cells and enhance wound healing. PLoS ONE.

[7] Maguire G. (2013). Stem cell therapy without the cells. Commun. Integr. Biol..

[8] Raposo G., Stoorvogel W. (2013). Extracellular vesicles: Exosomes, microvesicles, and friends. J. Cell Biol..

[9] Théry C., Witwer K.W., Aikawa E., Alcaraz M.J., Anderson J.D., Andriantsitohaina R., Antoniou A., Arab T., Archer F., Atkin-Smith G.K. (2018). Minimal information for studies of extracellular vesicles 2018 (MISEV2018): A position statement of the International Society for Extracellular Vesicles and update of the MISEV2014 guidelines. J. Extracell. Vesicles.

[10] De Jong O.G., Van Balkom B.W., Schiffelers R.M., Bouten C.V., Verhaar M.C. (2014). Extracellular vesicles: Potential roles in regenerative medicine. Front. Immunol..

[11] Brunet A., Goodell M.A., Rando T.A. (2022). Ageing and rejuvenation of tissue stem cells and their niches. Nat. Rev. Mol. Cell Biol..

[12] López-Otín C., Blasco M.A., Partridge L., Serrano M., Kroemer G. (2013). The hallmarks of aging. Cell.

[13] Kennedy B.K., Berger S.L., Brunet A., Campisi J., Cuervo A.M., Epel E.S., Franceschi C., Lithgow G.J., Morimoto R.I., Pessin J.E. (2014). Geroscience: Linking aging to chronic disease. Cell.

[14] Campisi J., d’Adda di Fagagna F. (2007). Cellular senescence: When bad things happen to good cells. Nat. Rev. Mol. Cell Biol..

[15] Collado M., Blasco M.A., Serrano M. (2007). Cellular senescence in cancer and aging. Cell.

[16] Coppé J.P., Desprez P.Y., Krtolica A., Campisi J. (2010). The senescence-associated secretory phenotype: The dark side of tumor suppression. Annu. Rev. Pathol..

[17] Tchkonia T., Zhu Y., van Deursen J., Campisi J., Kirkland J.L. (2013). Cellular senescence and the senescent secretory phenotype: Therapeutic opportunities. J. Clin. Investig..

[18] Fulzele S., Mendhe B., Khayrullin A., Johnson M., Kaiser H., Liu Y., Isales C.M., Hamrick M.W. (2019). Muscle-derived miR-34a increases with age in circulating extracellular vesicles and induces senescence of bone marrow stem cells. Aging.

[19] Borghesan M., Fafián-Labora J., Eleftheriadou O., Carpintero-Fernández P., Paez-Ribes M., Vizcay-Barrena G., Swisa A., Kolodkin-Gal D., Ximénez-Embún P., Lowe R. (2019). Small Extracellular Vesicles Are Key Regulators of Non-cell Autonomous Intercellular Communication in Senescence via the Interferon Protein IFITM3. Cell Rep..

[20] Fafián-Labora J.A., O’Loghlen A. (2021). NF-κB/IKK activation by small extracellular vesicles within the SASP. Aging Cell.

[21] Misawa T., Tanaka Y., Okada R., Takahashi A. (2020). Biology of extracellular vesicles secreted from senescent cells as senescence-associated secretory phenotype factors. Geriatr. Gerontol. Int..

[22] Mas-Bargues C., Sanz-Ros J., Román-Domínguez A., Gimeno-Mallench L., Inglés M., Viña J., Borrás C. (2020). Extracellular Vesicles from Healthy Cells Improves Cell Function and Stemness in Premature Senescent Stem Cells by miR-302b and HIF-1α Activation. Biomolecules.

[23] Fafián-Labora J.A., Rodríguez-Navarro J.A., O’Loghlen A. (2020). Small Extracellular Vesicles Have GST Activity and Ameliorate Senescence-Related Tissue Damage. Cell Metab..

[24] Sanz-Ros J., Romero-García N., Mas-Bargues C., Monleón D., Gordevicius J., Brooke R.T., Dromant M., Díaz A., Derevyanko A., Guío-Carrión A. (2022). Small extracellular vesicles from young adipose-derived stem cells prevent frailty, improve health span, and decrease epigenetic age in old mice. Sci. Adv..

[25] Yoshida M., Satoh A., Lin J.B., Mills K.F., Sasaki Y., Rensing N., Wong M., Apte R.S., Imai S.I. (2019). Extracellular Vesicle-Contained eNAMPT Delays Aging and Extends Lifespan in Mice. Cell Metab..

[26] Dorronsoro A., Santiago F.E., Grassi D., Zhang T., Lai R.C., McGowan S.J., Angelini L., Lavasani M., Corbo L., Lu A. (2021). Mesenchymal stem cell-derived extracellular vesicles reduce senescence and extend health span in mouse models of aging. Aging Cell.

[27] Harman D. (1956). Aging: A theory based on free radical and radiation chemistry. J. Gerontol..

[28] Viña J., Borras C., Abdelaziz K.M., Garcia-Valles R., Gomez-Cabrera M.C. (2013). The free radical theory of aging revisited: The cell signaling disruption theory of aging. Antioxid. Redox Signal..

[29] Gella A., Durany N. (2009). Oxidative stress in Alzheimer disease. Cell Adhes. Migr..

[30] Viña J., Borras C., Gomez-Cabrera M.C. (2018). A free radical theory of frailty. Free. Radic. Biol. Med..

[31] Inglés M., Gambini J., Carnicero J.A., García-García F.J., Rodríguez-Mañas L., Olaso-González G., Dromant M., Borrás C., Viña J. (2014). Oxidative stress is related to frailty, not to age or sex, in a geriatric population: Lipid and protein oxidation as biomarkers of frailty. J. Am. Geriatr. Soc..

[32] Borras C., Mas-Bargues C., Sanz-Ros J., Roman-Dominguez A., Gimeno-Mallench L., Ingles M., Gambini J., Vina J. (2020). Extracellular vesicles and redox modulation in aging. Free Radic. Biol. Med..

[33] Mas-Bargues C., Sanz-Ros J., Roman-Dominguez A., Ingles M., Gimeno-Mallench L., El Alami M., Vina-Almunia J., Gambini J., Vina J., Borras C. (2019). Relevance of Oxygen Concentration in Stem Cell Culture for Regenerative Medicine. Int. J. Mol. Sci..

[34] Mas-Bargues C., Borrás C. (2021). Importance of stem cell culture conditions for their derived extracellular vesicles therapeutic effect. Free Radic. Biol. Med..

[35] Han Y.D., Bai Y., Yan X.L., Ren J., Zeng Q., Li X.D., Pei X.T., Han Y. (2018). Co-transplantation of exosomes derived from hypoxia-preconditioned adipose mesenchymal stem cells promotes neovascularization and graft survival in fat grafting. Biochem. Biophys. Res. Commun..

[36] Cui G.H., Wu J., Mou F.F., Xie W.H., Wang F.B., Wang Q.L., Fang J., Xu Y.W., Dong Y.R., Liu J.R. (2018). Exosomes derived from hypoxia-preconditioned mesenchymal stromal cells ameliorate cognitive decline by rescuing synaptic dysfunction and regulating inflammatory responses in APP/PS1 mice. FASEB J..

[37] Blasco M.A. (2007). Telomere length, stem cells and aging. Nat. Chem. Biol..

[38] Whittemore K., Vera E., Martínez-Nevado E., Sanpera C., Blasco M.A. (2019). Telomere shortening rate predicts species life span. Proc. Natl. Acad. Sci. USA.

[39] Wang Z., Deng Z., Dahmane N., Tsai K., Wang P., Williams D.R., Kossenkov A.V., Showe L.C., Zhang R., Huang Q. (2015). Telomeric repeat-containing RNA (TERRA) constitutes a nucleoprotein component of extracellular inflammatory exosomes. Proc. Natl. Acad. Sci. USA.

[40] Al-Mayah A.H., Bright S.J., Bowler D.A., Slijepcevic P., Goodwin E., Kadhim M.A. (2017). Exosome-Mediated Telomere Instability in Human Breast Epithelial Cancer Cells after X Irradiation. Radiat. Res..

[41] Lanna A., Vaz B., D’Ambra C., Valvo S., Vuotto C., Chiurchiù V., Devine O., Sanchez M., Borsellino G., Akbar A.N. (2022). An intercellular transfer of telomeres rescues T cells from senescence and promotes long-term immunological memory. Nat. Cell Biol..

[42] Cuervo A.M. (2008). Autophagy and aging: Keeping that old broom working. Trends Genet..

[43] Kaushik S., Tasset I., Arias E., Pampliega O., Wong E., Martinez-Vicente M., Cuervo A.M. (2021). Autophagy and the hallmarks of aging. Ageing Res. Rev..

[44] Kiriyama Y., Nochi H. (2015). The Function of Autophagy in Neurodegenerative Diseases. Int. J. Mol. Sci..

[45] Guo H., Chitiprolu M., Roncevic L., Javalet C., Hemming F.J., Trung M.T., Meng L., Latreille E., Tanese de Souza C., McCulloch D. (2017). Atg5 Disassociates the V_1_V_0_-ATPase to Promote Exosome Production and Tumor Metastasis Independent of Canonical Macroautophagy. Dev. Cell.

[46] Jin J., Shi Y., Gong J., Zhao L., Li Y., He Q., Huang H. (2019). Exosome secreted from adipose-derived stem cells attenuates diabetic nephropathy by promoting autophagy flux and inhibiting apoptosis in podocyte. Stem Cell Res. Ther..

[47] Ebrahim N., Ahmed I.A., Hussien N.I., Dessouky A.A., Farid A.S., Elshazly A.M., Mostafa O., Gazzar W.B.E., Sorour S.M., Seleem Y. (2018). Mesenchymal Stem Cell-Derived Exosomes Ameliorated Diabetic Nephropathy by Autophagy Induction through the mTOR Signaling Pathway. Cells.

[48] Qu Y., Zhang Q., Cai X., Li F., Ma Z., Xu M., Lu L. (2017). Exosomes derived from miR-181-5p-modified adipose-derived mesenchymal stem cells prevent liver fibrosis via autophagy activation. J. Cell. Mol. Med..

[49] Rong Y., Liu W., Wang J., Fan J., Luo Y., Li L., Kong F., Chen J., Tang P., Cai W. (2019). Neural stem cell-derived small extracellular vesicles attenuate apoptosis and neuroinflammation after traumatic spinal cord injury by activating autophagy. Cell Death Dis..

[50] Buzas E.I., György B., Nagy G., Falus A., Gay S. (2014). Emerging role of extracellular vesicles in inflammatory diseases. Nat. Rev. Rheumatol..

[51] Ryan S.T., Hosseini-Beheshti E., Afrose D., Ding X., Xia B., Grau G.E., Little C.B., McClements L., Li J.J. (2021). Extracellular Vesicles from Mesenchymal Stromal Cells for the Treatment of Inflammation-Related Conditions. Int. J. Mol. Sci..

[52] Bruno S., Deregibus M.C., Camussi G. (2015). The secretome of mesenchymal stromal cells: Role of extracellular vesicles in immunomodulation. Immunol. Lett..

[53] Gao F., Chiu S.M., Motan D.A., Zhang Z., Chen L., Ji H.L., Tse H.F., Fu Q.L., Lian Q. (2016). Mesenchymal stem cells and immunomodulation: Current status and future prospects. Cell Death Dis..

[54] Franceschi C., Campisi J. (2014). Chronic inflammation (inflammaging) and its potential contribution to age-associated diseases. J. Gerontol. A Biol. Sci. Med. Sci..

[55] Xia S., Zhang X., Zheng S., Khanabdali R., Kalionis B., Wu J., Wan W., Tai X. (2016). An Update on Inflamm-Aging: Mechanisms, Prevention, and Treatment. J. Immunol. Res..

[56] Ferrucci L., Fabbri E. (2018). Inflammageing: Chronic inflammation in ageing, cardiovascular disease, and frailty. Nat. Rev. Cardiol..

[57] Wang W., Wang L., Ruan L., Oh J., Dong X., Zhuge Q., Su D.M. (2018). Extracellular vesicles extracted from young donor serum attenuate inflammaging via partially rejuvenating aged T-cell immunotolerance. FASEB J..

[58] Lee B.R., Kim J.H., Choi E.S., Cho J.H., Kim E. (2018). Effect of young exosomes injected in aged mice. Int. J. Nanomed..

[59] Junnila R.K., List E.O., Berryman D.E., Murrey J.W., Kopchick J.J. (2013). The GH/IGF-1 axis in ageing and longevity. Nat. Rev. Endocrinol..

[60] Kenyon C.J. (2010). The genetics of ageing. Nature.

[61] Fontana L., Partridge L., Longo V.D. (2010). Extending healthy life span—From yeast to humans. Science.

[62] Barzilai N., Huffman D.M., Muzumdar R.H., Bartke A. (2012). The critical role of metabolic pathways in aging. Diabetes.

[63] Johnson S.C., Rabinovitch P.S., Kaeberlein M. (2013). mTOR is a key modulator of ageing and age-related disease. Nature.

[64] Harrison D.E., Strong R., Sharp Z.D., Nelson J.F., Astle C.M., Flurkey K., Nadon N.L., Wilkinson J.E., Frenkel K., Carter C.S. (2009). Rapamycin fed late in life extends lifespan in genetically heterogeneous mice. Nature.

[65] Colman R.J., Anderson R.M., Johnson S.C., Kastman E.K., Kosmatka K.J., Beasley T.M., Allison D.B., Cruzen C., Simmons H.A., Kemnitz J.W. (2009). Caloric restriction delays disease onset and mortality in rhesus monkeys. Science.

[66] Castaño C., Kalko S., Novials A., Párrizas M. (2018). Obesity-associated exosomal miRNAs modulate glucose and lipid metabolism in mice. Proc. Natl. Acad. Sci. USA.

[67] Deng Z.B., Poliakov A., Hardy R.W., Clements R., Liu C., Liu Y., Wang J., Xiang X., Zhang S., Zhuang X. (2009). Adipose tissue exosome-like vesicles mediate activation of macrophage-induced insulin resistance. Diabetes.

[68] Zhang H., Jin K. (2020). Peripheral Circulating Exosomal miRNAs Potentially Contribute to the Regulation of Molecular Signaling Networks in Aging. Int. J. Mol. Sci..

[69] Turovsky E.A., Golovicheva V.V., Varlamova E.G., Danilina T.I., Goryunov K.V., Shevtsova Y.A., Pevzner I.B., Zorova L.D., Babenko V.A., Evtushenko E.A. (2022). Mesenchymal stromal cell-derived extracellular vesicles afford neuroprotection by modulating PI3K/AKT pathway and calcium oscillations. Int. J. Biol. Sci..

[70] Li C., Jiao G., Wu W., Wang H., Ren S., Zhang L., Zhou H., Liu H., Chen Y. (2019). Exosomes from Bone Marrow Mesenchymal Stem Cells Inhibit Neuronal Apoptosis and Promote Motor Function Recovery via the Wnt/β-catenin Signaling Pathway. Cell Transpl..

[71] Arslan F., Lai R.C., Smeets M.B., Akeroyd L., Choo A., Aguor E.N., Timmers L., van Rijen H.V., Doevendans P.A., Pasterkamp G. (2013). Mesenchymal stem cell-derived exosomes increase ATP levels, decrease oxidative stress and activate PI3K/Akt pathway to enhance myocardial viability and prevent adverse remodeling after myocardial ischemia/reperfusion injury. Stem Cell Res..

[72] Bian S., Zhang L., Duan L., Wang X., Min Y., Yu H. (2014). Extracellular vesicles derived from human bone marrow mesenchymal stem cells promote angiogenesis in a rat myocardial infarction model. J. Mol. Med..

[73] Todorova D., Simoncini S., Lacroix R., Sabatier F., Dignat-George F. (2017). Extracellular Vesicles in Angiogenesis. Circ. Res..

[74] Choi J.S., Yoon H.I., Lee K.S., Choi Y.C., Yang S.H., Kim I.S., Cho Y.W. (2016). Exosomes from differentiating human skeletal muscle cells trigger myogenesis of stem cells and provide biochemical cues for skeletal muscle regeneration. J. Control Release.

[75] Sahu A., Clemens Z.J., Shinde S.N., Sivakumar S., Pius A., Bhatia A., Picciolini S., Carlomagno C., Gualerzi A., Bedoni M. (2021). Regulation of aged skeletal muscle regeneration by circulating extracellular vesicles. Nat. Aging.

[76] Zuo R., Liu M., Wang Y., Li J., Wang W., Wu J., Sun C., Li B., Wang Z., Lan W. (2019). BM-MSC-derived exosomes alleviate radiation-induced bone loss by restoring the function of recipient BM-MSCs and activating Wnt/β-catenin signaling. Stem Cell Res. Ther..

[77] Liang B., Liang J.M., Ding J.N., Xu J., Xu J.G., Chai Y.M. (2019). Dimethyloxaloylglycine-stimulated human bone marrow mesenchymal stem cell-derived exosomes enhance bone regeneration through angiogenesis by targeting the AKT/mTOR pathway. Stem Cell Res. Ther..

[78] Wei X., Yi X., Lv H., Sui X., Lu P., Li L., An Y., Yang Y., Yi H., Chen G. (2020). MicroRNA-377-3p released by mesenchymal stem cell exosomes ameliorates lipopolysaccharide-induced acute lung injury by targeting RPTOR to induce autophagy. Cell Death Dis..

[79] Kadota T., Fujita Y., Araya J., Watanabe N., Fujimoto S., Kawamoto H., Minagawa S., Hara H., Ohtsuka T., Yamamoto Y. (2021). Human bronchial epithelial cell-derived extracellular vesicle therapy for pulmonary fibrosis via inhibition of TGF-β-WNT crosstalk. J. Extracell. Vesicles.

[80] Shen B., Liu J., Zhang F., Wang Y., Qin Y., Zhou Z., Qiu J., Fan Y. (2016). CCR2 Positive Exosome Released by Mesenchymal Stem Cells Suppresses Macrophage Functions and Alleviates Ischemia/Reperfusion-Induced Renal Injury. Stem Cells Int..

[81] Pan G.Z., Yang Y., Zhang J., Liu W., Wang G.Y., Zhang Y.C., Yang Q., Zhai F.X., Tai Y., Liu J.R. (2012). Bone marrow mesenchymal stem cells ameliorate hepatic ischemia/reperfusion injuries via inactivation of the MEK/ERK signaling pathway in rats. J. Surg. Res..

[82] Steward M.M., Sridhar A., Meyer J.S. (2013). Neural regeneration. New Perspectives in Regeneration.

[83] Ma Y., Dong L., Zhou D., Li L., Zhang W., Zhen Y., Wang T., Su J., Chen D., Mao C. (2019). Extracellular vesicles from human umbilical cord mesenchymal stem cells improve nerve regeneration after sciatic nerve transection in rats. J. Cell. Mol. Med..

[84] Ma Y., Ge S., Zhang J., Zhou D., Li L., Wang X., Su J. (2017). Mesenchymal stem cell-derived extracellular vesicles promote nerve regeneration after sciatic nerve crush injury in rats. Int. J. Clin. Exp. Pathol..

[85] Zhang Y., Chopp M., Meng Y., Katakowski M., Xin H., Mahmood A., Xiong Y. (2015). Effect of exosomes derived from multipluripotent mesenchymal stromal cells on functional recovery and neurovascular plasticity in rats after traumatic brain injury. J. Neurosurg..

[86] Xin H., Li Y., Buller B., Katakowski M., Zhang Y., Wang X., Shang X., Zhang Z.G., Chopp M. (2012). Exosome-mediated transfer of miR-133b from multipotent mesenchymal stromal cells to neural cells contributes to neurite outgrowth. Stem Cells.

[87] Xin H., Li Y., Cui Y., Yang J.J., Zhang Z.G., Chopp M. (2013). Systemic administration of exosomes released from mesenchymal stromal cells promote functional recovery and neurovascular plasticity after stroke in rats. J. Cereb. Blood Flow Metab..

[88] Sisa C., Kholia S., Naylor J., Herrera Sanchez M.B., Bruno S., Deregibus M.C., Camussi G., Inal J.M., Lange S., Hristova M. (2019). Mesenchymal Stromal Cell Derived Extracellular Vesicles Reduce Hypoxia-Ischaemia Induced Perinatal Brain Injury. Front. Physiol..

[89] Bucan V., Vaslaitis D., Peck C.T., Strauß S., Vogt P.M., Radtke C. (2019). Effect of Exosomes from Rat Adipose-Derived Mesenchymal Stem Cells on Neurite Outgrowth and Sciatic Nerve Regeneration After Crush Injury. Mol. Neurobiol..

[90] Lai R.C., Arslan F., Lee M.M., Sze N.S., Choo A., Chen T.S., Salto-Tellez M., Timmers L., Lee C.N., El Oakley R.M. (2010). Exosome secreted by MSC reduces myocardial ischemia/reperfusion injury. Stem Cell Res..

[91] He L., Nguyen N.B., Ardehali R., Zhou B. (2020). Heart Regeneration by Endogenous Stem Cells and Cardiomyocyte Proliferation: Controversy, Fallacy, and Progress. Circulation.

[92] Zhu L.P., Tian T., Wang J.Y., He J.N., Chen T., Pan M., Xu L., Zhang H.X., Qiu X.T., Li C.C. (2018). Hypoxia-elicited mesenchymal stem cell-derived exosomes facilitates cardiac repair through miR-125b-mediated prevention of cell death in myocardial infarction. Theranostics.

[93] Han C., Zhou J., Liang C., Liu B., Pan X., Zhang Y., Wang Y., Yan B., Xie W., Liu F. (2019). Human umbilical cord mesenchymal stem cell derived exosomes encapsulated in functional peptide hydrogels promote cardiac repair. Biomater. Sci..

[94] Mastrullo V., Cathery W., Velliou E., Madeddu P., Campagnolo P. (2020). Angiogenesis in Tissue Engineering: As Nature Intended?. Front. Bioeng. Biotechnol..

[95] Nakamura Y., Miyaki S., Ishitobi H., Matsuyama S., Nakasa T., Kamei N., Akimoto T., Higashi Y., Ochi M. (2015). Mesenchymal-stem-cell-derived exosomes accelerate skeletal muscle regeneration. FEBS Lett..

[96] Figliolini F., Ranghino A., Grange C., Cedrino M., Tapparo M., Cavallari C., Rossi A., Togliatto G., Femminò S., Gugliuzza M.V. (2020). Extracellular Vesicles From Adipose Stem Cells Prevent Muscle Damage and Inflammation in a Mouse Model of Hind Limb Ischemia: Role of Neuregulin-1. Arterioscler. Thromb. Vasc. Biol..

[97] Wang C., Song W., Chen B., Liu X., He Y. (2019). Exosomes Isolated From Adipose-Derived Stem Cells: A New Cell-Free Approach to Prevent the Muscle Degeneration Associated With Torn Rotator Cuffs. Am. J. Sports Med..

[98] Furuta T., Miyaki S., Ishitobi H., Ogura T., Kato Y., Kamei N., Miyado K., Higashi Y., Ochi M. (2016). Mesenchymal Stem Cell-Derived Exosomes Promote Fracture Healing in a Mouse Model. Stem Cells Transl. Med..

[99] Takeuchi R., Katagiri W., Endo S., Kobayashi T. (2019). Exosomes from conditioned media of bone marrow-derived mesenchymal stem cells promote bone regeneration by enhancing angiogenesis. PLoS ONE.

[100] Zhang S., Chuah S.J., Lai R.C., Hui J.H.P., Lim S.K., Toh W.S. (2018). MSC exosomes mediate cartilage repair by enhancing proliferation, attenuating apoptosis and modulating immune reactivity. Biomaterials.

[101] Willis G.R., Fernandez-Gonzalez A., Reis M., Yeung V., Liu X., Ericsson M., Andrews N.A., Mitsialis S.A., Kourembanas S. (2020). Mesenchymal stromal cell-derived small extracellular vesicles restore lung architecture and improve exercise capacity in a model of neonatal hyperoxia-induced lung injury. J. Extracell. Vesicles.

[102] Monsel A., Zhu Y.G., Gennai S., Hao Q., Hu S., Rouby J.J., Rosenzwajg M., Matthay M.A., Lee J.W. (2015). Therapeutic Effects of Human Mesenchymal Stem Cell-derived Microvesicles in Severe Pneumonia in Mice. Am. J. Respir. Crit. Care Med..

[103] Zhu Y.G., Feng X.M., Abbott J., Fang X.H., Hao Q., Monsel A., Qu J.M., Matthay M.A., Lee J.W. (2014). Human mesenchymal stem cell microvesicles for treatment of Escherichia coli endotoxin-induced acute lung injury in mice. Stem Cells.

[104] Li J.W., Wei L., Han Z., Chen Z. (2019). Mesenchymal stromal cells-derived exosomes alleviate ischemia/reperfusion injury in mouse lung by transporting anti-apoptotic miR-21-5p. Eur. J. Pharmacol..

[105] Denic A., Glassock R.J., Rule A.D. (2016). Structural and Functional Changes With the Aging Kidney. Adv. Chronic Kidney Dis..

[106] Berger K., Bangen J.M., Hammerich L., Liedtke C., Floege J., Smeets B., Moeller M.J. (2014). Origin of regenerating tubular cells after acute kidney injury. Proc. Natl. Acad. Sci. USA.

[107] Bruno S., Grange C., Deregibus M.C., Calogero R.A., Saviozzi S., Collino F., Morando L., Busca A., Falda M., Bussolati B. (2009). Mesenchymal stem cell-derived microvesicles protect against acute tubular injury. J. Am. Soc. Nephrol..

[108] Zhou Y., Liu S., Zhao M., Wang C., Li L., Yuan Y., Liao G., Bresette W., Zhang J., Chen Y. (2019). Injectable extracellular vesicle-released self-assembling peptide nanofiber hydrogel as an enhanced cell-free therapy for tissue regeneration. J. Control Release.

[109] He J., Wang Y., Sun S., Yu M., Wang C., Pei X., Zhu B., Wu J., Zhao W. (2012). Bone marrow stem cells-derived microvesicles protect against renal injury in the mouse remnant kidney model. Nephrology.

[110] Bruno S., Grange C., Collino F., Deregibus M.C., Cantaluppi V., Biancone L., Tetta C., Camussi G. (2012). Microvesicles derived from mesenchymal stem cells enhance survival in a lethal model of acute kidney injury. PLoS ONE.

[111] Ramírez-Bajo M.J., Martín-Ramírez J., Bruno S., Pasquino C., Banon-Maneus E., Rovira J., Moya-Rull D., Lazo-Rodriguez M., Campistol J.M., Camussi G. (2020). Nephroprotective Potential of Mesenchymal Stromal Cells and Their Extracellular Vesicles in a Murine Model of Chronic Cyclosporine Nephrotoxicity. Front. Cell Dev. Biol..

[112] Tan C.Y., Lai R.C., Wong W., Dan Y.Y., Lim S.K., Ho H.K. (2014). Mesenchymal stem cell-derived exosomes promote hepatic regeneration in drug-induced liver injury models. Stem Cell Res. Ther..

[113] Nong K., Wang W., Niu X., Hu B., Ma C., Bai Y., Wu B., Wang Y., Ai K. (2016). Hepatoprotective effect of exosomes from human-induced pluripotent stem cell-derived mesenchymal stromal cells against hepatic ischemia-reperfusion injury in rats. Cytotherapy.

[114] Yao J., Zheng J., Cai J., Zeng K., Zhou C., Zhang J., Li S., Li H., Chen L., He L. (2019). Extracellular vesicles derived from human umbilical cord mesenchymal stem cells alleviate rat hepatic ischemia-reperfusion injury by suppressing oxidative stress and neutrophil inflammatory response. FASEB J..

[115] Bruno S., Chiabotto G., Camussi G. (2020). Extracellular Vesicles: A Therapeutic Option for Liver Fibrosis. Int. J. Mol. Sci..

[116] Sun Z., Hou X., Zhang J., Li J., Wu P., Yan L., Qian H. (2022). Diagnostic and Therapeutic Roles of Extracellular Vesicles in Aging-Related Diseases. Oxid. Med. Cell. Longev..

[117] Das M., Kale V. (2021). Involvement of extracellular vesicles in aging process and their beneficial effects in alleviating aging-associated symptoms. Cell Biol. Int..

[118] Yin Y., Chen H., Wang Y., Zhang L., Wang X. (2021). Roles of extracellular vesicles in the aging microenvironment and age-related diseases. J. Extracell. Vesicles.

[119] Apodaca L.A., Baddour A.A.D., Garcia C., Alikhani L., Giedzinski E., Ru N., Agrawal A., Acharya M.M., Baulch J.E. (2021). Human neural stem cell-derived extracellular vesicles mitigate hallmarks of Alzheimer’s disease. Alzheimers Res. Ther..

[120] Chen Y.A., Lu C.H., Ke C.C., Chiu S.J., Jeng F.S., Chang C.W., Yang B.H., Liu R.S. (2021). Mesenchymal Stem Cell-Derived Exosomes Ameliorate Alzheimer’s Disease Pathology and Improve Cognitive Deficits. Biomedicines.

[121] Li B., Liu J., Gu G., Han X., Zhang Q., Zhang W. (2020). Impact of neural stem cell-derived extracellular vesicles on mitochondrial dysfunction, sirtuin 1 level, and synaptic deficits in Alzheimer’s disease. J. Neurochem..

[122] Losurdo M., Pedrazzoli M., D’Agostino C., Elia C.A., Massenzio F., Lonati E., Mauri M., Rizzi L., Molteni L., Bresciani E. (2020). Intranasal delivery of mesenchymal stem cell-derived extracellular vesicles exerts immunomodulatory and neuroprotective effects in a 3xTg model of Alzheimer’s disease. Stem Cells Transl. Med..

[123] Zhai L., Shen H., Sheng Y., Guan Q. (2021). ADMSC Exo-MicroRNA-22 improve neurological function and neuroinflammation in mice with Alzheimer’s disease. J. Cell. Mol. Med..

[124] Wang J.C., Bennett M. (2012). Aging and atherosclerosis: Mechanisms, functional consequences, and potential therapeutics for cellular senescence. Circ. Res..

[125] Libby P., Buring J.E., Badimon L., Hansson G.K., Deanfield J., Bittencourt M.S., Tokgözoğlu L., Lewis E.F. (2019). Atherosclerosis. Nat. Rev. Dis. Prim..

[126] Ma J., Chen L., Zhu X., Li Q., Hu L., Li H. (2021). Mesenchymal stem cell-derived exosomal miR-21a-5p promotes M2 macrophage polarization and reduces macrophage infiltration to attenuate atherosclerosis. Acta Biochim. Biophys. Sin..

[127] Li J., Tan M., Xiang Q., Zhou Z., Yan H. (2017). Thrombin-activated platelet-derived exosomes regulate endothelial cell expression of ICAM-1 via microRNA-223 during the thrombosis-inflammation response. Thromb. Res..

[128] Li J., Xue H., Li T., Chu X., Xin D., Xiong Y., Qiu W., Gao X., Qian M., Xu J. (2019). Exosomes derived from mesenchymal stem cells attenuate the progression of atherosclerosis in ApoE. Biochem. Biophys. Res. Commun..

[129] Gao H., Yu Z., Li Y., Wang X. (2021). miR-100-5p in human umbilical cord mesenchymal stem cell-derived exosomes mediates eosinophilic inflammation to alleviate atherosclerosis via the FZD5/Wnt/β-catenin pathway. Acta Biochim. Biophys. Sin..

[130] Chen S., Zhou H., Zhang B., Hu Q. (2021). Exosomal miR-512-3p derived from mesenchymal stem cells inhibits oxidized low-density lipoprotein-induced vascular endothelial cells dysfunction via regulating Keap1. J. Biochem. Mol. Toxicol..

[131] Bai S., Yin Q., Dong T., Dai F., Qin Y., Ye L., Du J., Zhang Q., Chen H., Shen B. (2020). Endothelial progenitor cell-derived exosomes ameliorate endothelial dysfunction in a mouse model of diabetes. Biomed. Pharmacother..

[132] Longo M., Bellastella G., Maiorino M.I., Meier J.J., Esposito K., Giugliano D. (2019). Diabetes and Aging: From Treatment Goals to Pharmacologic Therapy. Front. Endocrinol..

[133] Sun Y., Shi H., Yin S., Ji C., Zhang X., Zhang B., Wu P., Shi Y., Mao F., Yan Y. (2018). Human Mesenchymal Stem Cell Derived Exosomes Alleviate Type 2 Diabetes Mellitus by Reversing Peripheral Insulin Resistance and Relieving β-Cell Destruction. ACS Nano.

[134] He Q., Wang L., Zhao R., Yan F., Sha S., Cui C., Song J., Hu H., Guo X., Yang M. (2020). Mesenchymal stem cell-derived exosomes exert ameliorative effects in type 2 diabetes by improving hepatic glucose and lipid metabolism via enhancing autophagy. Stem Cell Res. Ther..

[135] Zhao H., Shang Q., Pan Z., Bai Y., Li Z., Zhang H., Zhang Q., Guo C., Zhang L., Wang Q. (2018). Exosomes From Adipose-Derived Stem Cells Attenuate Adipose Inflammation and Obesity Through Polarizing M2 Macrophages and Beiging in White Adipose Tissue. Diabetes.

[136] Cantaluppi V., Biancone L., Figliolini F., Beltramo S., Medica D., Deregibus M.C., Galimi F., Romagnoli R., Salizzoni M., Tetta C. (2012). Microvesicles derived from endothelial progenitor cells enhance neoangiogenesis of human pancreatic islets. Cell Transpl..

[137] Sun Y., Mao Q., Shen C., Wang C., Jia W. (2019). Exosomes from β-cells alleviated hyperglycemia and enhanced angiogenesis in islets of streptozotocin-induced diabetic mice. Diabetes Metab. Syndr. Obes..

[138] Ji M.X., Yu Q. (2015). Primary osteoporosis in postmenopausal women. Chronic Dis. Transl. Med..

[139] Zhang X., Wang W., Wang Y., Zhao H., Han X., Zhao T., Qu P. (2020). Extracellular Vesicle-Encapsulated miR-29b-3p Released From Bone Marrow-Derived Mesenchymal Stem Cells Underpins Osteogenic Differentiation. Front. Cell Dev. Biol..

[140] Yang X., Yang J., Lei P., Wen T. (2019). LncRNA MALAT1 shuttled by bone marrow-derived mesenchymal stem cells-secreted exosomes alleviates osteoporosis through mediating microRNA-34c/SATB2 axis. Aging.

[141] Song H., Li X., Zhao Z., Qian J., Wang Y., Cui J., Weng W., Cao L., Chen X., Hu Y. (2019). Reversal of Osteoporotic Activity by Endothelial Cell-Secreted Bone Targeting and Biocompatible Exosomes. Nano Lett..

[142] Qiu M., Zhai S., Fu Q., Liu D. (2021). Bone Marrow Mesenchymal Stem Cells-Derived Exosomal MicroRNA-150-3p Promotes Osteoblast Proliferation and Differentiation in Osteoporosis. Hum. Gene Ther..

[143] Zhang X., Wang Y., Zhao H., Han X., Zhao T., Qu P., Li G., Wang W. (2020). Extracellular vesicle-encapsulated miR-22-3p from bone marrow mesenchymal stem cell promotes osteogenic differentiation via FTO inhibition. Stem Cell Res. Ther..

[144] Lu G.D., Cheng P., Liu T., Wang Z. (2020). BMSC-Derived Exosomal miR-29a Promotes Angiogenesis and Osteogenesis. Front. Cell Dev. Biol..

[145] Hu Y., Zhang Y., Ni C.Y., Chen C.Y., Rao S.S., Yin H., Huang J., Tan Y.J., Wang Z.X., Cao J. (2020). Human umbilical cord mesenchymal stromal cells-derived extracellular vesicles exert potent bone protective effects by CLEC11A-mediated regulation of bone metabolism. Theranostics.

[146] Loeser R.F., Collins J.A., Diekman B.O. (2016). Ageing and the pathogenesis of osteoarthritis. Nat. Rev. Rheumatol..

[147] Loeser R.F. (2011). Aging and osteoarthritis. Curr. Opin. Rheumatol..

[148] Zhu C., Wu W., Qu X. (2021). Mesenchymal stem cells in osteoarthritis therapy: A review. Am. J. Transl. Res..

[149] Woo C.H., Kim H.K., Jung G.Y., Jung Y.J., Lee K.S., Yun Y.E., Han J., Lee J., Kim W.S., Choi J.S. (2020). Small extracellular vesicles from human adipose-derived stem cells attenuate cartilage degeneration. J. Extracell. Vesicles.

[150] Wang Y., Yu D., Liu Z., Zhou F., Dai J., Wu B., Zhou J., Heng B.C., Zou X.H., Ouyang H. (2017). Exosomes from embryonic mesenchymal stem cells alleviate osteoarthritis through balancing synthesis and degradation of cartilage extracellular matrix. Stem Cell Res. Ther..

[151] Zhang S., Teo K.Y.W., Chuah S.J., Lai R.C., Lim S.K., Toh W.S. (2019). MSC exosomes alleviate temporomandibular joint osteoarthritis by attenuating inflammation and restoring matrix homeostasis. Biomaterials.

[152] Gorgun C., Palamà M.E.F., Reverberi D., Gagliani M.C., Cortese K., Tasso R., Gentili C. (2021). Role of extracellular vesicles from adipose tissue- and bone marrow-mesenchymal stromal cells in endothelial proliferation and chondrogenesis. Stem Cells Transl. Med..

[153] Ragni E., Perucca Orfei C., De Luca P., Colombini A., Viganò M., de Girolamo L. (2020). Secreted Factors and EV-miRNAs Orchestrate the Healing Capacity of Adipose Mesenchymal Stem Cells for the Treatment of Knee Osteoarthritis. Int. J. Mol. Sci..

[154] Jin Z., Ren J., Qi S. (2020). Human bone mesenchymal stem cells-derived exosomes overexpressing microRNA-26a-5p alleviate osteoarthritis via down-regulation of PTGS2. Int. Immunopharmacol..

[155] Shimoda A., Sawada S.I., Sasaki Y., Akiyoshi K. (2019). Exosome surface glycans reflect osteogenic differentiation of mesenchymal stem cells: Profiling by an evanescent field fluorescence-assisted lectin array system. Sci. Rep..

[156] O’Sullivan E.D., Hughes J., Ferenbach D.A. (2017). Renal Aging: Causes and Consequences. J. Am. Soc. Nephrol..

[157] Kholia S., Herrera Sanchez M.B., Cedrino M., Papadimitriou E., Tapparo M., Deregibus M.C., Bruno S., Antico F., Brizzi M.F., Quesenberry P.J. (2020). Mesenchymal Stem Cell Derived Extracellular Vesicles Ameliorate Kidney Injury in Aristolochic Acid Nephropathy. Front. Cell Dev. Biol..

[158] He J., Wang Y., Lu X., Zhu B., Pei X., Wu J., Zhao W. (2015). Micro-vesicles derived from bone marrow stem cells protect the kidney both in vivo and in vitro by microRNA-dependent repairing. Nephrology.

[159] Shi Z., Wang Q., Zhang Y., Jiang D. (2020). Extracellular vesicles produced by bone marrow mesenchymal stem cells attenuate renal fibrosis, in part by inhibiting the RhoA/ROCK pathway, in a UUO rat model. Stem Cell Res. Ther..

[160] Grange C., Tritta S., Tapparo M., Cedrino M., Tetta C., Camussi G., Brizzi M.F. (2019). Stem cell-derived extracellular vesicles inhibit and revert fibrosis progression in a mouse model of diabetic nephropathy. Sci. Rep..

[161] Yu L., Liu S., Wang C., Zhang C., Wen Y., Zhang K., Chen S., Huang H., Liu Y., Wu L. (2021). Embryonic stem cell-derived extracellular vesicles promote the recovery of kidney injury. Stem Cell Res. Ther..

[162] Fried L.P., Tangen C.M., Walston J., Newman A.B., Hirsch C., Gottdiener J., Seeman T., Tracy R., Kop W.J., Burke G. (2001). Frailty in older adults: Evidence for a phenotype. J. Gerontol. A Biol. Sci. Med. Sci..

[163] Chen X., Mao G., Leng S.X. (2014). Frailty syndrome: An overview. Clin. Interv. Aging.

[164] Partridge L., Deelen J., Slagboom P.E. (2018). Facing up to the global challenges of ageing. Nature.

[165] Seals D.R., Melov S. (2014). Translational geroscience: Emphasizing function to achieve optimal longevity. Aging.

[166] van Niel G., Carter D.R.F., Clayton A., Lambert D.W., Raposo G., Vader P. (2022). Challenges and directions in studying cell-cell communication by extracellular vesicles. Nat. Rev. Mol. Cell Biol..

[167] Rezaie J., Feghhi M., Etemadi T. (2022). A review on exosomes application in clinical trials: Perspective, questions, and challenges. Cell Commun. Signal..

[168] Gimona M., Pachler K., Laner-Plamberger S., Schallmoser K., Rohde E. (2017). Manufacturing of Human Extracellular Vesicle-Based Therapeutics for Clinical Use. Int. J. Mol. Sci..

[169] Herrmann I.K., Wood M.J.A., Fuhrmann G. (2021). Extracellular vesicles as a next-generation drug delivery platform. Nat. Nanotechnol..

[170] Chevillet J.R., Kang Q., Ruf I.K., Briggs H.A., Vojtech L.N., Hughes S.M., Cheng H.H., Arroyo J.D., Meredith E.K., Gallichotte E.N. (2014). Quantitative and stoichiometric analysis of the microRNA content of exosomes. Proc. Natl. Acad. Sci. USA.

[171] Albanese M., Chen Y.A., Hüls C., Gärtner K., Tagawa T., Mejias-Perez E., Keppler O.T., Göbel C., Zeidler R., Shein M. (2021). MicroRNAs are minor constituents of extracellular vesicles that are rarely delivered to target cells. PLoS Genet..

